# Author Correction: GWAS of thyroid stimulating hormone highlights the pleiotropic effects and inverse association with thyroid cancer

**DOI:** 10.1038/s41467-021-27675-w

**Published:** 2021-12-16

**Authors:** Wei Zhou, Ben Brumpton, Omer Kabil, Julius Gudmundsson, Gudmar Thorleifsson, Josh Weinstock, Matthew Zawistowski, Jonas B. Nielsen, Layal Chaker, Marco Medici, Alexander Teumer, Silvia Naitza, Serena Sanna, Ulla T. Schultheiss, Anne Cappola, Juha Karjalainen, Mitja Kurki, Morgan Oneka, Peter Taylor, Lars G. Fritsche, Sarah E. Graham, Brooke N. Wolford, William Overton, Humaira Rasheed, Eirin B. Haug, Maiken E. Gabrielsen, Anne Heidi Skogholt, Ida Surakka, George Davey Smith, Anita Pandit, Tanmoy Roychowdhury, Whitney E. Hornsby, Jon G. Jonasson, Leigha Senter, Sandya Liyanarachchi, Matthew D. Ringel, Li Xu, Lambertus A. Kiemeney, Huiling He, Romana T. Netea-Maier, Jose I. Mayordomo, Theo S. Plantinga, Jon Hrafnkelsson, Hannes Hjartarson, Erich M. Sturgis, Aarno Palotie, Mark Daly, Cintia E. Citterio, Peter Arvan, Chad M. Brummett, Michael Boehnke, Albert de la Chapelle, Kari Stefansson, Kristian Hveem, Cristen J. Willer, Bjørn Olav Åsvold

**Affiliations:** 1 Department of Computational Medicine and Bioinformatics, University of Michigan, Ann Arbor, Michigan USA; 2 Analytic and Translational Genetics Unit, Massachusetts General Hospital, Boston, Massachusetts USA; 3 Program in Medical and Population Genetics, Broad Institute of Harvard and MIT, Cambridge, Massachusetts USA; 4 Stanley Center for Psychiatric Research, Broad Institute of Harvard and MIT, Cambridge, Massachusetts USA; 5 K.G. Jebsen Center for Genetic Epidemiology, Department of Public Health and Nursing, NTNU, Norwegian University of Science and Technology, Trondheim, Norway; 6 Medical Research Council (MRC) Integrative Epidemiology Unit, University of Bristol, Bristol, UK; 7 Department of Thoracic Medicine, St. Olavs Hospital, Trondheim University Hospital, Trondheim, Norway; 8 Department of Biological Chemistry, University of Michigan Medical School, Ann Arbor, Michigan USA; 9 Division of Metabolism Endocrinology and Diabetes, University of Michigan Medical School, Ann Arbor, Michigan USA; 10 deCODE genetics/AMGEN, 101 Reykjavik, Iceland; 11 Center for Statistical Genetics and Department of Biostatistics, University of Michigan School of Public Health, Ann Arbor, Michigan USA; 12 Department of Internal Medicine, Division of Cardiology, University of Michigan Medical School, Ann Arbor, Michigan USA; 13 Department of Epidemiology Research, Statens Serum Institute, Copenhagen, Denmark; 14 Erasmus MC Academic Center for Thyroid Diseases, Rotterdam, The Netherlands; 15 Department of Epidemiology, Erasmus Medical Center, Rotterdam, The Netherlands; 16 Department of Internal Medicine, Erasmus Medical Center, Rotterdam, The Netherlands; 17 Division of Endocrinology, Department of Internal Medicine, Radboud University Medical Centre, Radboud Institute for Molecular Life Sciences, 6500HB Nijmegen, The Netherlands; 18 Institute for Community Medicine, University Medicine Greifswald, Greifswald, Germany; 19 DZHK (German Center for Cardiovascular Research), Partner Site Greifswald, Greifswald, Germany; 20 Istituto di Ricerca Genetica e Biomedica, Consiglio Nazionale delle Ricerche Monserrato, Monserrato, Italy; 21 Department of Genetics, University of Groningen, University Medical Center Groningen, Groningen, The Netherlands; 22 Faculty of Medicine and Medical Center, Institute of Genetic Epidemiology, University of Freiburg, Freiburg, Germany; 23 Faculty of Medicine and Medical Center, Department of Medicine IV–Nephrology and Primary Care, University of Freiburg, Freiburg, Germany; 24 Division of Endocrinology, Diabetes, and Metabolism, University of Pennsylvania School of Medicine, Philadelphia, Pennsylvania USA; 25 Institute for Molecular Medicine Finland, Helsinki Institute of Life Sciences, University of Helsinki, Helsinki, 00014 Finland; 26 Thyroid Research Group, Systems Immunity Research Institute, Cardiff University School of Medicine, Cardiff, UK; 27 Faculty of Medicine and Health Sciences, Department of Public Health and Nursing, Norwegian University of Science and Technology, NTNU, Trondheim, Norway; 28 Department of Population Health Sciences, Bristol Medical School, University of Bristol, Bristol, UK; 29 Landspitali-University Hospital, 101 Reykjavik, Iceland; 30 Faculty of Medicine, University of Iceland, 101 Reykjavik, Iceland; 31 The Icelandic Cancer Registry, 105 Reykjavik, Iceland; 32 Division of Human Genetics, Ohio State University Comprehensive Cancer Center, Columbus, Ohio 43210 USA; 33 Department of Cancer Biology and Genetics, Ohio State University Comprehensive Cancer Center, Columbus, Ohio 43210 USA; 34 Division of Endocrinology, Diabetes, and Metabolism, The Ohio State University, Columbus, Ohio 43210 USA; 35 Department of Head and Neck Surgery, and Department of Epidemiology, The University of Texas MD Anderson Cancer Center, Houston, Texas 77030 USA; 36 Radboud University Medical Centre, Radboud Institute for Health Sciences, 6500HB Nijmegen, The Netherlands; 37 University of Colorado Hospital, Aurora, Colorado 80045 USA; 38 Department of Pathology, Radboud University Medical Center, Radboud Institute for Molecular Life Sciences, 6500HB Nijmegen, The Netherlands; 39 Universidad de Buenos Aires, Facultad de Farmacia y Bioquímica, Departamento de Microbiología, Inmunología y Biotecnología/Cátedra de Genética, Buenos Aires, C1113AAD Argentina; 40 CONICET-Universidad de Buenos Aires, Instituto de Inmunología, Genética y Metabolismo (INIGEM), C1120AAR Buenos Aires, Argentina; 41 Division of Pain Medicine, Department of Anesthesiology, University of Michigan Medical School, Ann Arbor, Michigan USA; 42 HUNT Research Centre, Department of Public Health and Nursing, NTNU, Norwegian University of Science and Technology, Levanger, 7600 Norway; 43 Department of Medicine, Levanger Hospital, Nord-Trøndelag Hospital Trust, Levanger, 7600 Norway; 44 Department of Human Genetics, University of Michigan, Ann Arbor, Michigan USA; 45 Department of Endocrinology, St. Olavs Hospital, Trondheim University Hospital, Trondheim, Norway

**Keywords:** Gene expression, Genome-wide association studies

Correction to: *Nature Communications* 10.1038/s41467-020-17718-z, published online 07 August 2020.

The original version of this article contained an error in the results, in the second paragraph of the subsection entitled “Fine-mapping for potentially causal variants among TSH loci”, in which effect sizes for two variants were incorrectly reported.

The original version incorrectly read ‘In the HUNT study, the missense variant *TG* p.G67S (rs116340633, MAF = 1.8%, effect size = 0.77 SD, 95% CI = 0.73–0.82 SD, *P*-value = 1.07 × 10^−21^) is in strong LD (*r*^2^ = 0.99) with the most strongly associated variant rs117074997 (intronic). At the other association signal, missense variant *TG* p.P118L (rs114322847, MAF = 2.4%, effect size = 0.84 SD, 95% CI = 0.82–0.87 SD, *P*-value = 1.87 × 10^−26^) is in strong LD (*r*^2^ = 0.92) with the most strongly associated variant rs118039499 (intronic) (Supplementary Table 2 and Supplementary Fig. 4)’.

The correct version replaces this sentence with ‘In the HUNT study, the missense variant *TG* p.G67S (rs116340633, MAF = 1.8%, effect size = −0.26 SD, 95% CI = −0.31 to −0.20 SD, *P*-value = 1.07 × 10^−21^) is in strong LD (*r*^2^ = 0.99) with the most strongly associated variant rs117074997 (intronic). At the other association signal, missense variant *TG* p.P118L (rs114322847, MAF = 2.4%, effect size = −0.17 SD, 95% CI = −0.20 to −0.14 SD, *P*-value = 1.87 × 10^−26^) is in strong LD (*r*^2^ = 0.92) with the most strongly associated variant rs118039499 (intronic) (Supplementary Table 2, Supplementary Figure 4)’.

This has been corrected in both the PDF and HTML versions of the Article.

