# Supplementary material for: GWAS of thyroid stimulating hormone highlights pleiotropic effects and inverse association with thyroid cancer
Source: Nat Commun. 2020 Aug 7;11:3981. doi: 10.1038/s41467-020-17718-z (PMC7414135; doi:10.1038/s41467-020-17718-z)
Supplement: Supplementary file 1 — Supplementary Information [file 41467_2020_17718_MOESM1_ESM.pdf]

GWAS of thyroid stimulating hormone highlights the pleiotropic effects and inverse association with thyroid cancer. Zhou *et al.*

Supplementary Figures

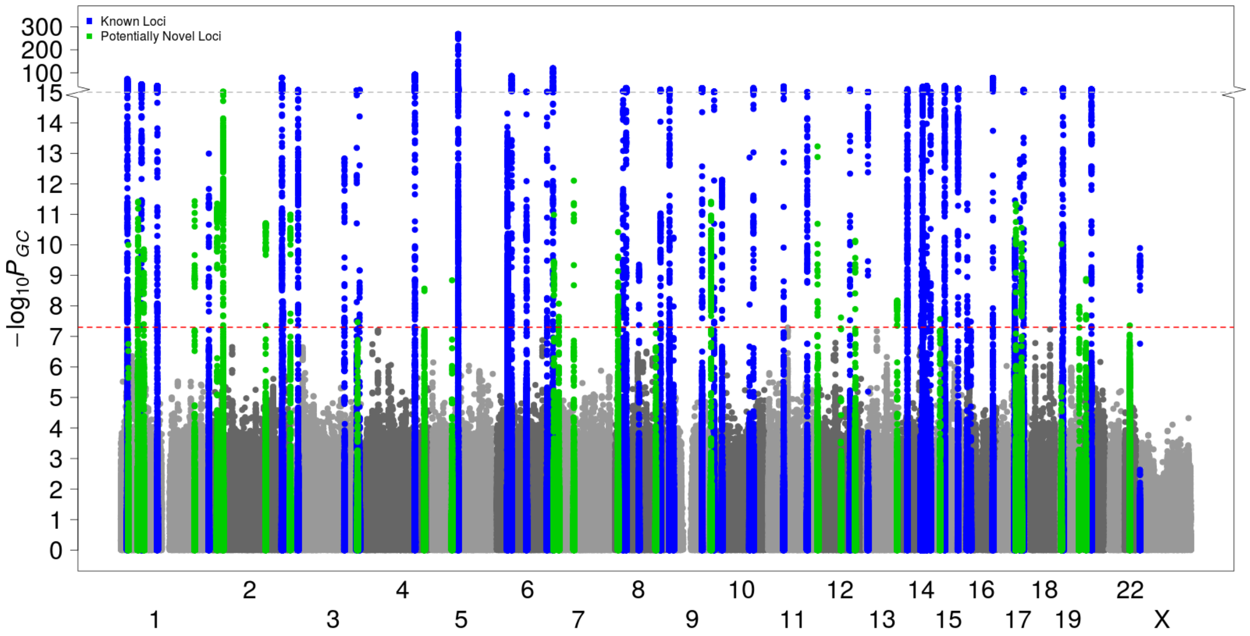

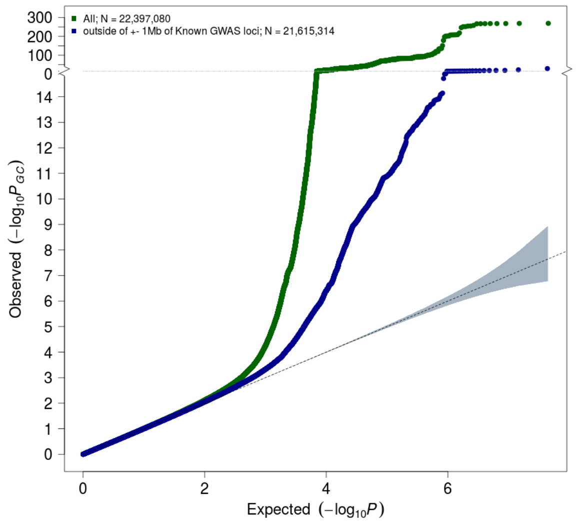

Supplementary Figure 1. Manhattan and Q-Q Plots for the meta-analysis GWAS of TSH from the HUNT study (N = 55,342), the MGI study (N = 10,085), and the ThyroidOmics consortium (N = up to 54,288). N: sample size.

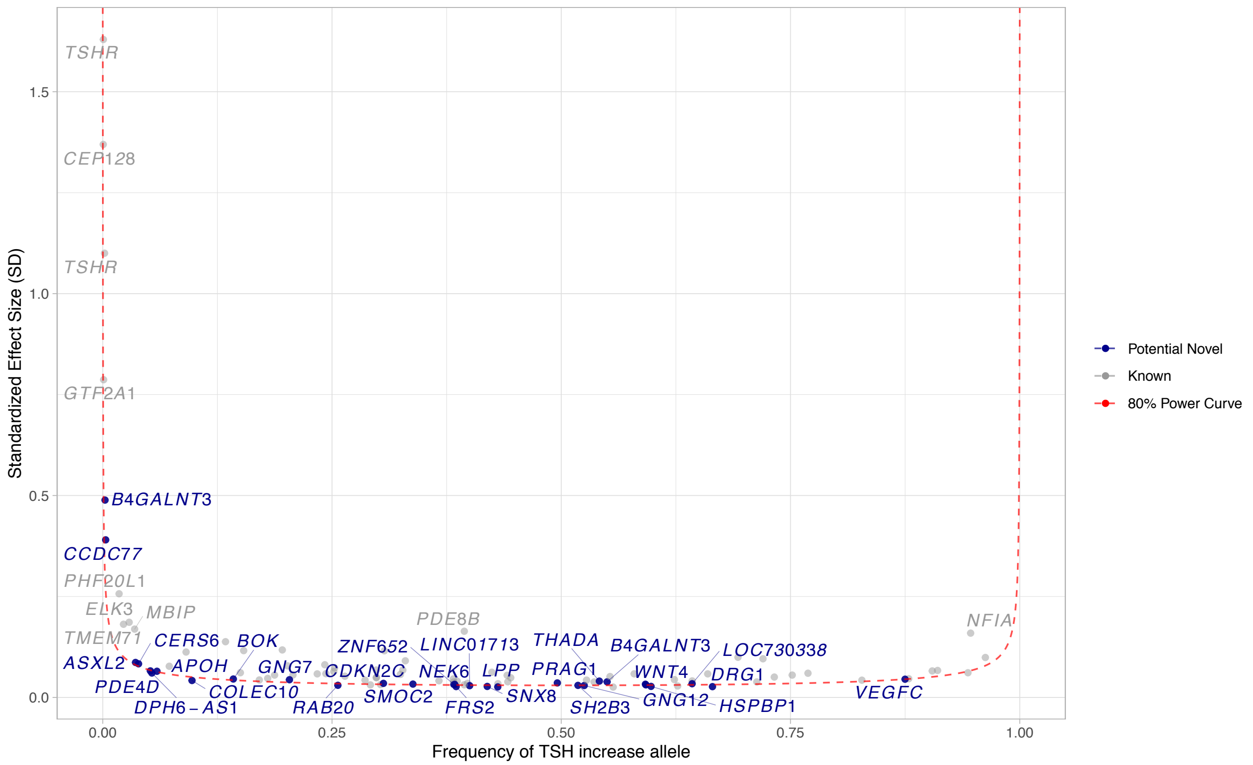

Supplementary Figure 2. A smile plot for the frequency of TSH increasing alleles versus the effect sizes for all 99 TSH associated variants**.** Novel TSH loci are highlighted in blue. Names of nearest genes for variants with abs(effect sizes) > 0.15 (in the unit of SD) are shown in the plot.

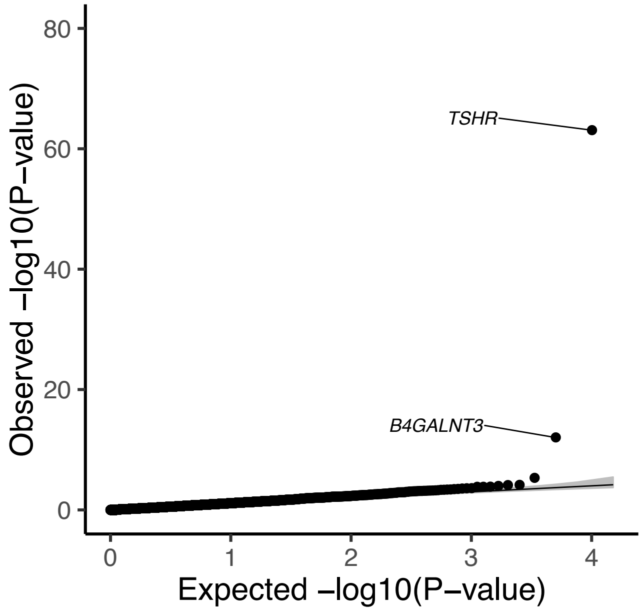

Supplementary Figure 3. Quantile-quantile plot for the gene-based SKAT-O test for TSH in HUNT.

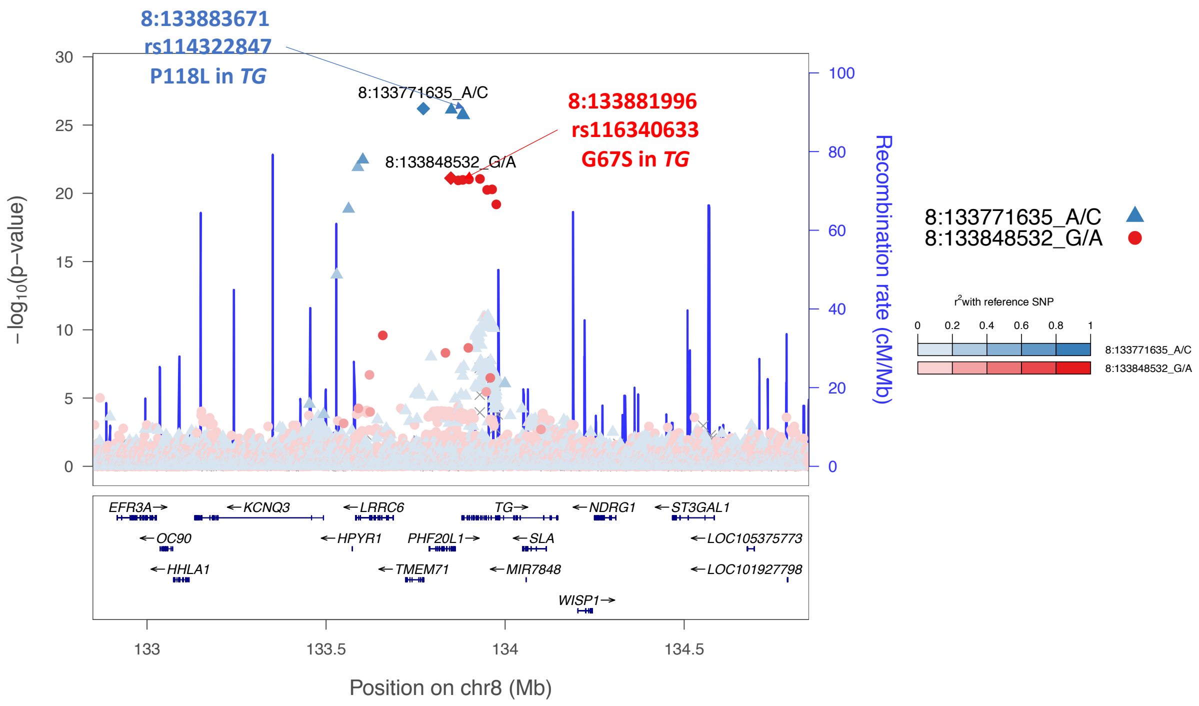

Supplementary Figure 4. Regional association plot for the locus 8q24.22 around the gene *TG* for the meta-analysis of HUNT, MGI and thyroidOmics, which harbors two independent association signals (LD r^2 between top variants, 8:133771635 and 8:133848532 = 0.0008). SNPs are colored according to their LD with the two top intronic variants (8:133771635 in blue and 8:133848532 in red). The LD between SNPs were calculated using dosages in the HUNT study.

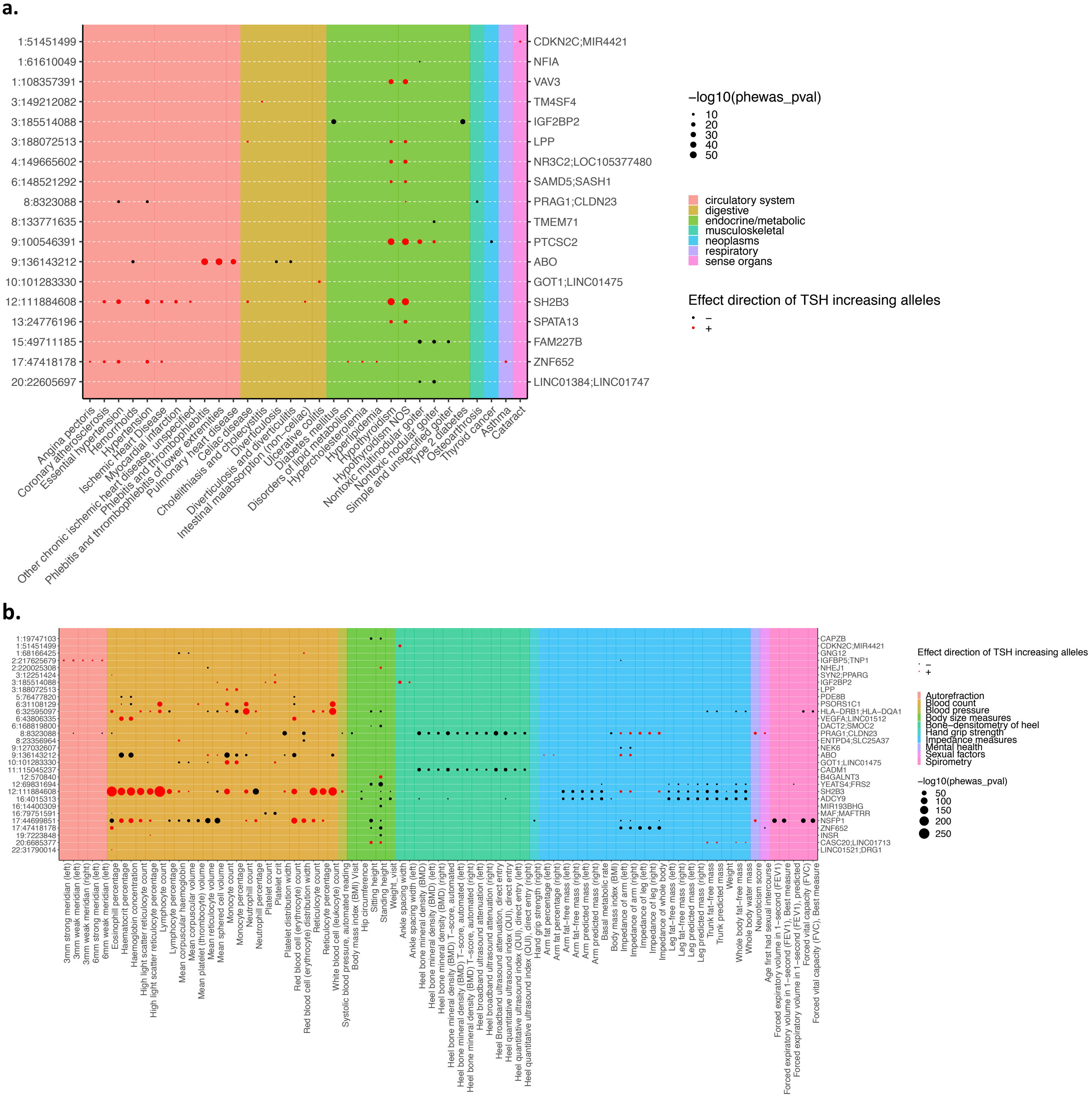

Supplementary Figure 5. a. TSH index variants are significantly associated (p-value < $5x10^{-8}$) with 1,283 human diseases in the UK Biobank b. TSH index variants are significantly associated (p-value < $5x10^{-10}$) with 274 quantitative traits in the UK Biobank.

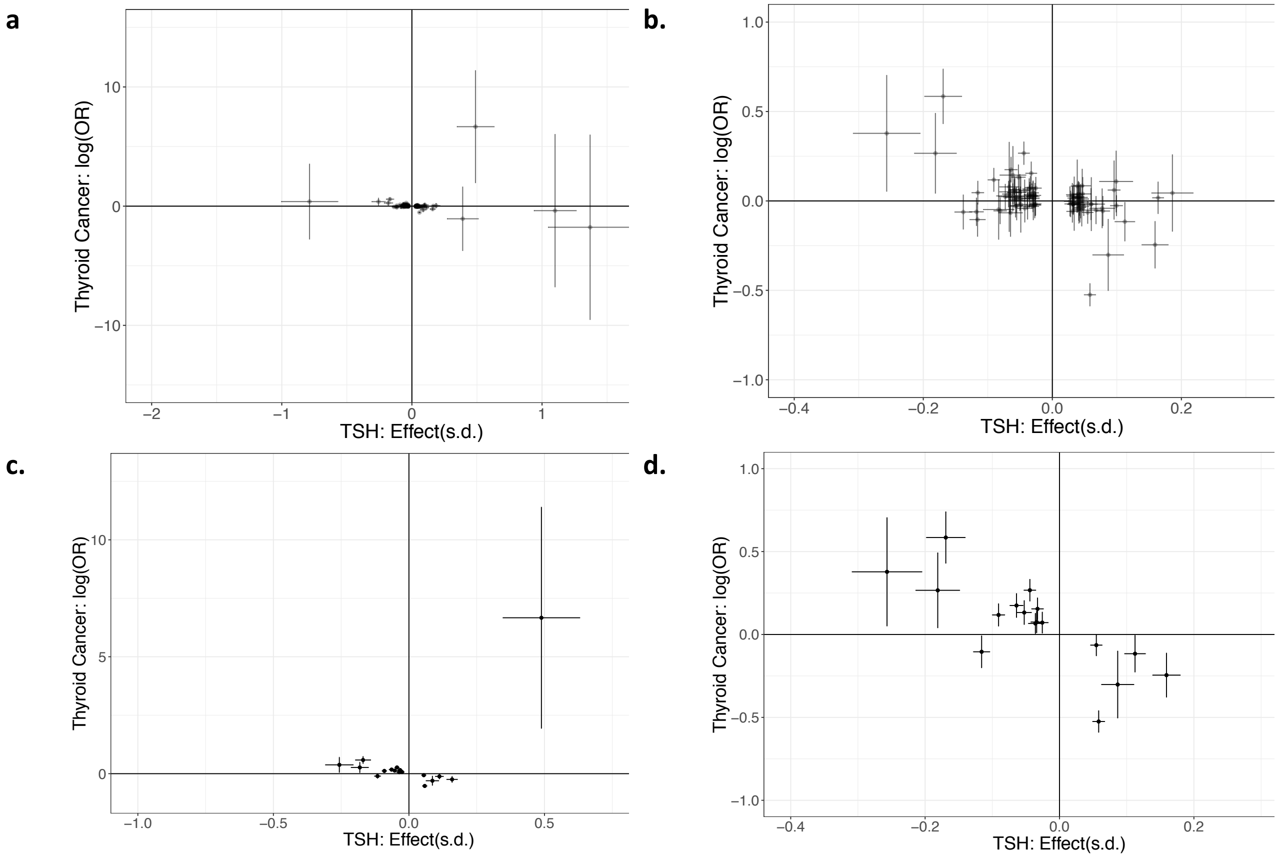

Supplementary Figure 6. plot of effects sizes (SD) for TSH and log of odds ratio (OR) for thyroid cancer of a. 94 TSH index variants (**Supplementary Data 9**), b. 89 out of 94 TSH index variants with MAF ≥ 1%, c. 18 TSH index variants with association p-value with thyroid cancer < 0.05 and d. 17 out of 18 index variants with MAF ≥ 1% (the rare variant rs145153320 in the gene *B4GALNT3* with MAF=0.00051 in UKBB and 0.0025 in HUNT was excluded from the plot).

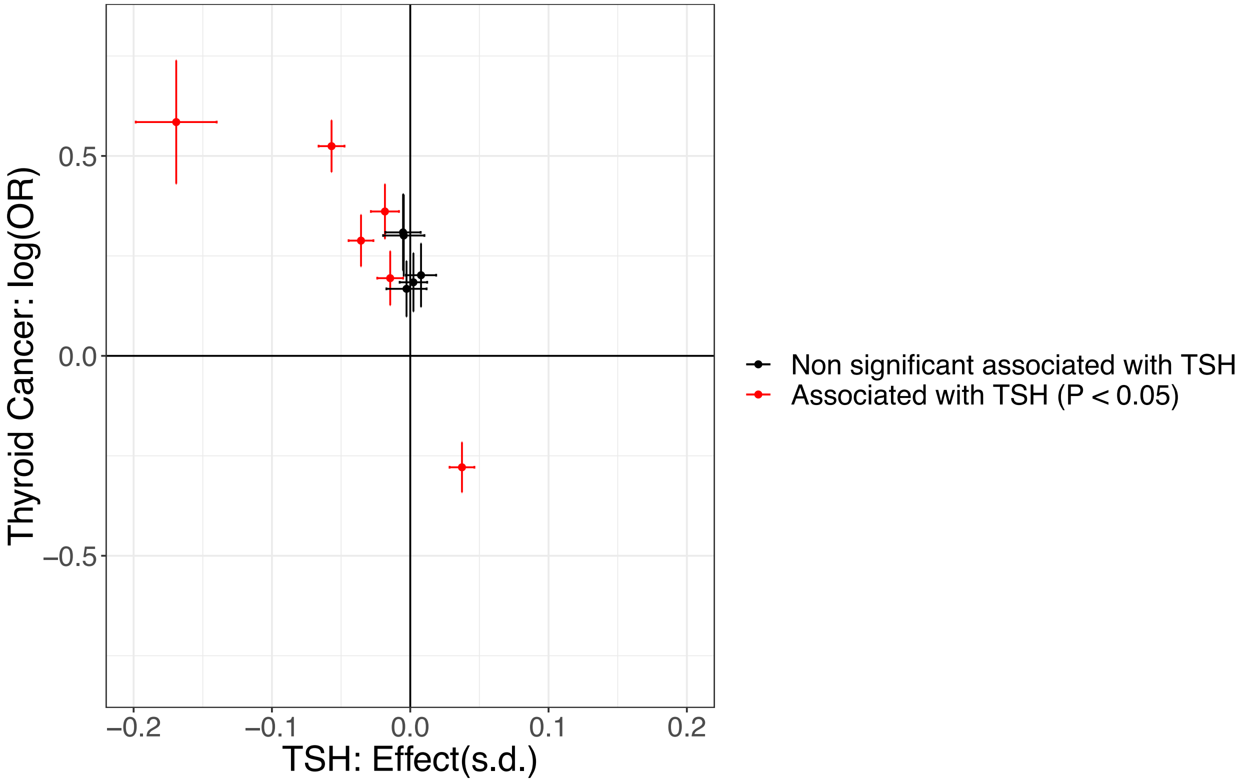

Supplementary Figure 7. plots of effects sizes (SD) for TSH and log of odds ratio (OR) for thyroid cancer of 11 previously identified variants for thyroid cancer (**Supplementary Data 10**).

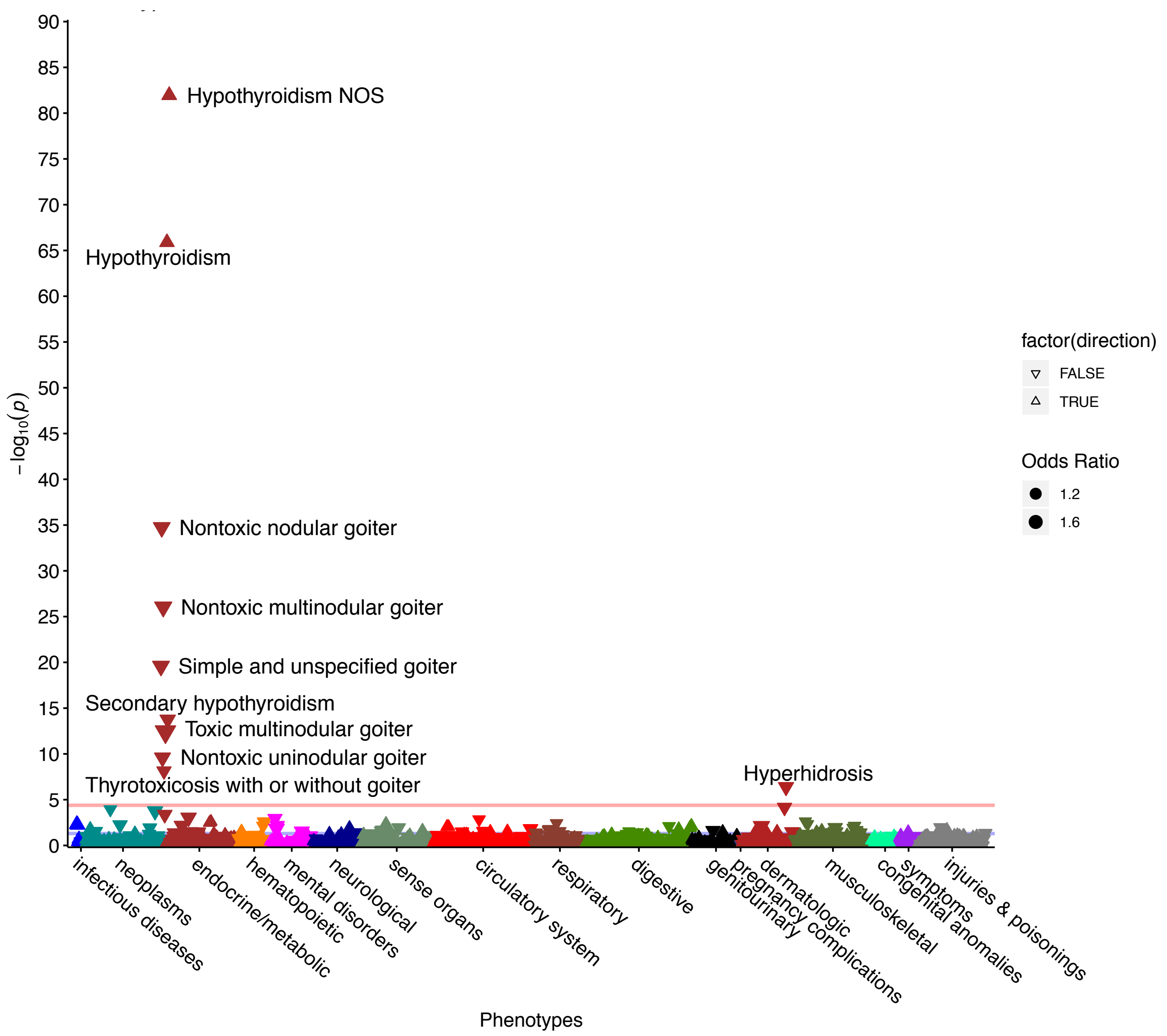

Supplementary Figure 8. Association results between TSH polygenic risk score and 1,283 binary phenotypes in UKB

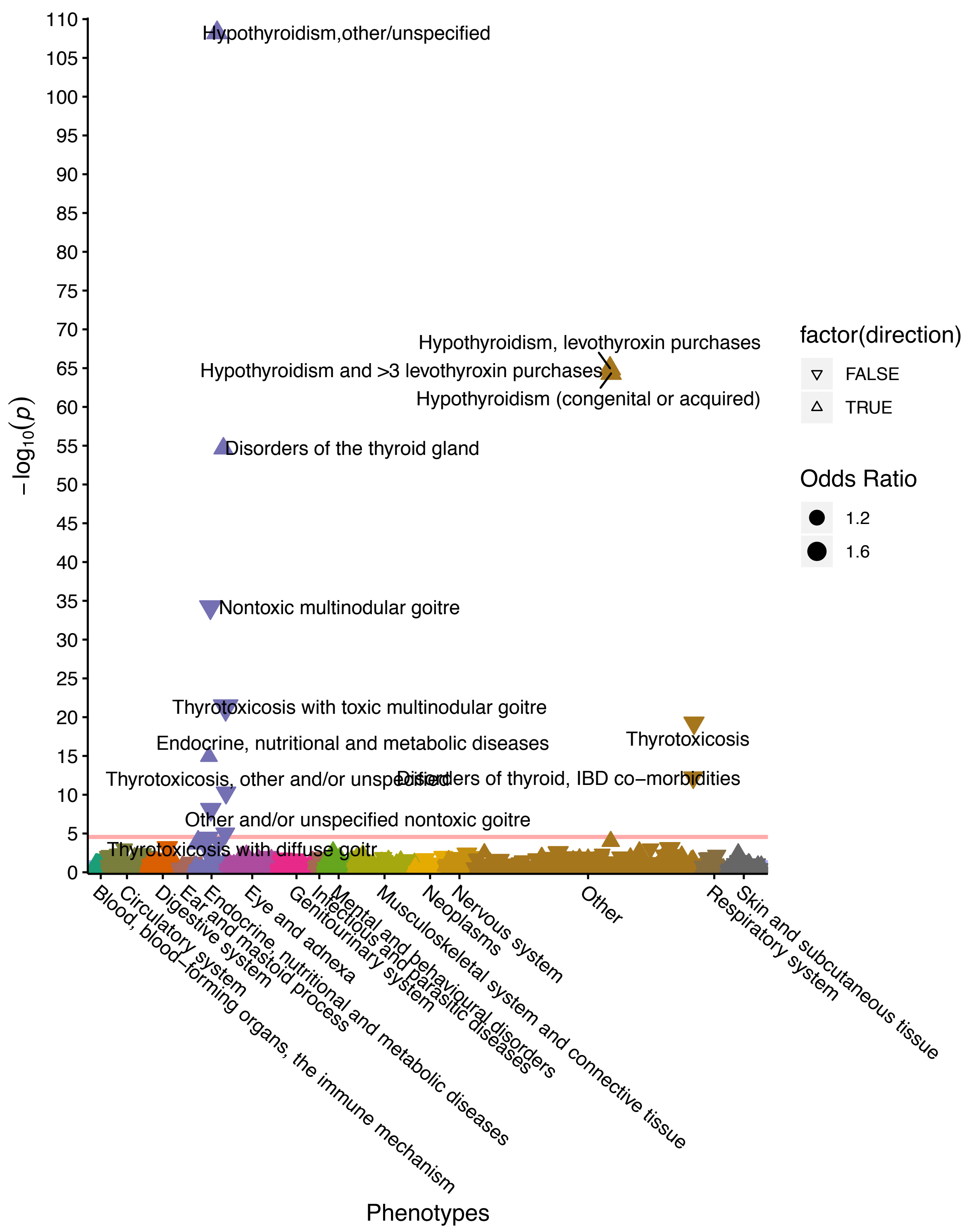

Supplementary Figure 9. Association results between TSH PGS and 1,801 binary phenotypes in FinnGen.

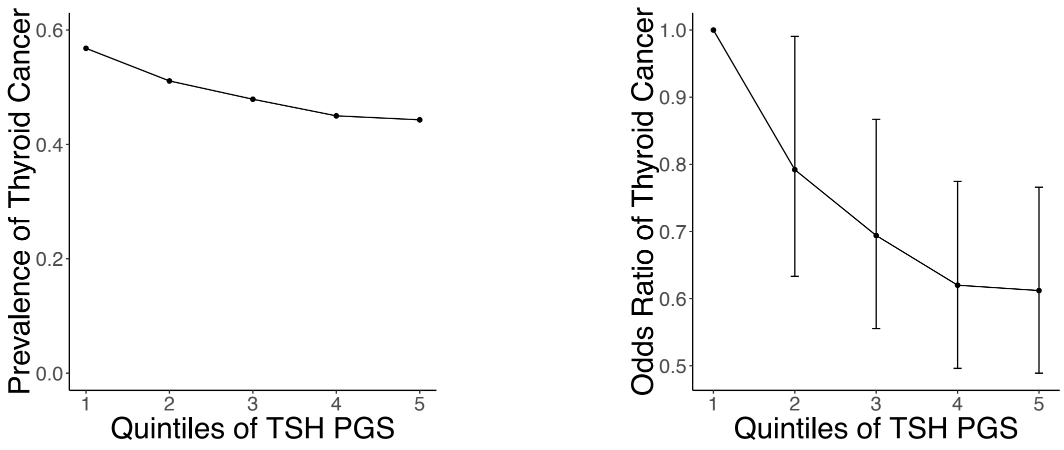

Supplementary Figure 10. The risk of thyroid cancer is lower for individuals with genetically predicted higher TSH levels for the Columbus, USA study. Plots of thyroid cancer prevalence by quintiles of TSH PGS (left panel) and odds ratio of thyroid cancer in relation to the lowest quintile (right panel) in the Columbus Ohio, USA study (N case=1,580, N control=1,628).N: sample size. N case: sample size of cases. N control: sample size of controls. Error bars represent 95% confidence intervals.

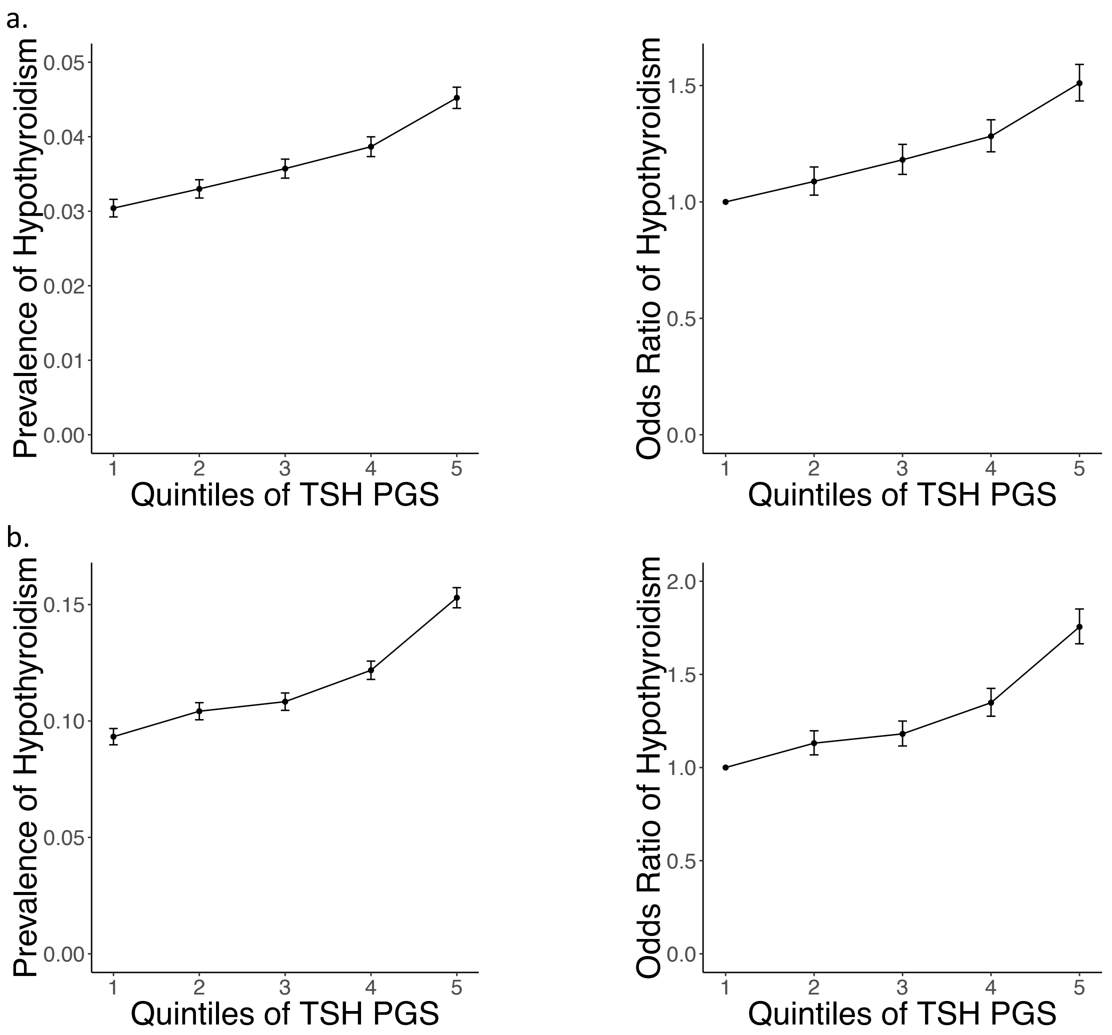

Supplementary Figure 11. The risk of Hypothyroidism is higher for individuals with genetically-higher TSH levels. Plots of hypothyroidism prevalence by quintiles of TSH PGS (left panel) and odds ratio of thyroid cancer in relation to the lowest quintile (right panel) in data sets a. UKBB (N case=14,871, N control=391,429), b. FinnGen(N case= 15,410, N control=117,331). N: sample size. N case: sample size of cases. N control: sample size of controls. Error bars represent 95% confidence intervals.

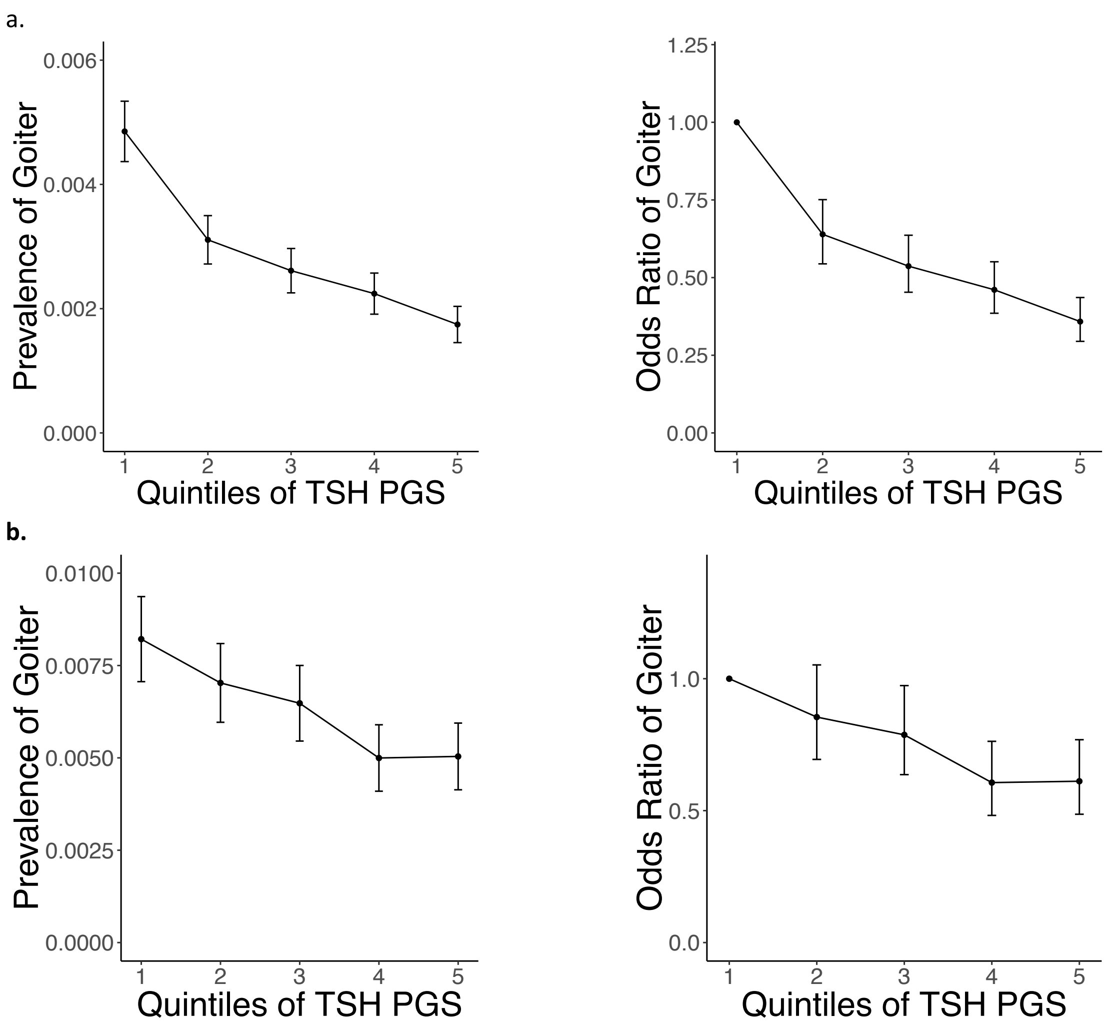

Supplementary Figure 12. The risk of Goiter is lower for individuals with genetically-higher TSH levels. Plots of goiter prevalence by quintiles of TSH PGS (left panel) and odds ratio of thyroid cancer in relation to the lowest quintile (right panel) in data sets a. UKBB (N case= 1,143, N control=391,429), b. FinnGen(N case=750, N control=117,331). N: sample size. N case: sample size of cases. N control: sample size of controls. Error bars represent 95% confidence intervals.

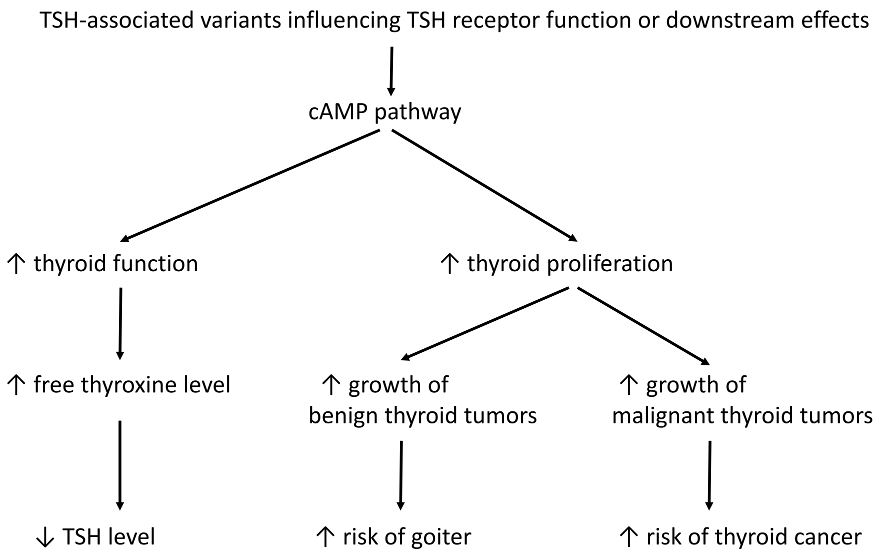

Supplementary Figure 13. Schematic presentation of potential pleiotropic effects of TSH-associated variants on TSH levels and risk of thyroid cancer and goiter.

a.

b.

c.

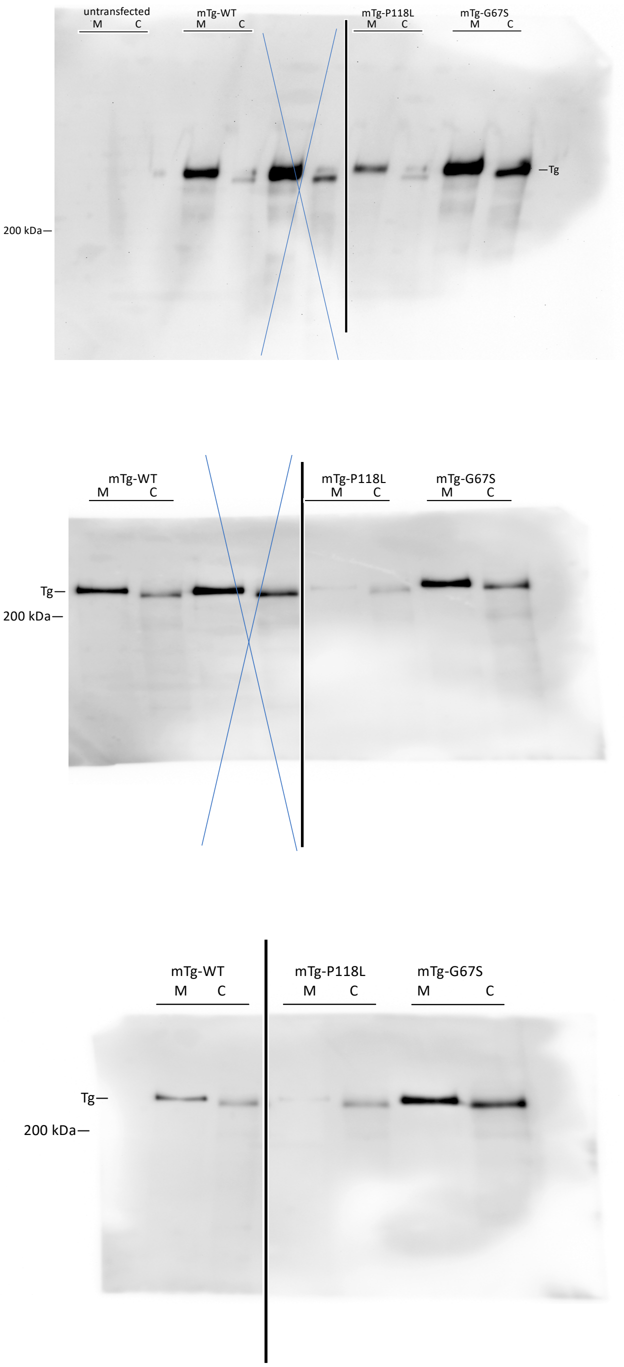

Supplementary Figure 14. Full scans of western blots. a. Western blot from **Figure 1a**. b. Western blot from **Figure 1b**. c. Western plot from **Figure 1c.** The parts that are unrelated to the manuscript in the full scans are marked as X.

Supplementary Table 1. Genes reached exome-wide significant threshold (P-value < $2.5X10^{-6}$) in the gene-based SKAT-O test with missense and stop-gain variants with MAF ≤ 0.5% and imputation quality score ≥ 0.8 by SAIGE-GENE in HUNT

Gene	Number of Markers	Variant IDs	Frequency of the alt allele	SKAT-O Test	SKAT-O Test	Top Hit in the Locus			SKAT-O Test	Second top hit in the Locus		
		chr:pos_ref/alt (GRCh37)		P-value	Conditional P-value (conditioning on the top hit)	Variant	MAF	P-value	Conditional P- value (conditioning on the two top hits)	Variant	MAF	P-value
TSHR	4	14:81422124_G/A;14:81557483_A/T;14:81610059_G/A;14:81610228_G/A	3.62e-05; 0.001; 0.0007; 0.002	$8.0x10^{-64}$	$2.04x10^{-30}$	chr14:81610228_G/A (rs139352934)	0.002	$2.66x10^{-41}$	$2.87x10^{-8}$	14:81610059_G/A (rs121908872)	0.001	$2.79x10^{-32}$
B4GALNT3	11	12:661265_C/T; 12:662597_C/T; 12:662605_A/G;12:662762_A/G;12:665814_G/A;12:665822_C/T;12:665955_G/A;12:665966_C/T;12:667217_G/A;12:667806_G/A;12:668548_G/A	0.0003; 6.25e-05; 9.14e-06; 0.0003; 3.47e-05; 0.0025; 2.78e-05; 0.00066; 1.84e-05; 0.00026; 0.0015	$8.90x10^{-13}$	0.3	chr12:665822_C/T (rs145153320)	0.0025	$1x10^{-11}$				

Supplementary Table 2. Two independent association signals were identified in the locus of the thyroglobulin gene TG. Each contains a missense variant that is in strong LD with the top hit. Genetics variants with association P-value < $5x10^{-8}$ in the meta-analysis of HUNT, MGI and ThyroidOmics that have LD r2 ≥ 0.2 with any one of the top hits in the 8q24.22 locus are included in the table

							**Meta-analysis of HUNT, MGI and ThyroidOmics**						
**Cluster**	**Position(GRCh37)**	**rsID**	**Ref**	**Alt**	**Allele frequency****	**Amino Acid Change**	**Effect***	**SE of Effect*****	**P-value**	**Function**	**Nearest Gene**	**r2 with chr8:133848532**	**r2 with chr8:133771635**
	chr8:133621394	rs770676596	C	T	0.005		-0.286	0.055	2.04E-07	intronic	*LRRC6*	0.221	0.000
	chr8:133658606	rs563535087	C	T	0.015		-0.197	0.031	2.59E-10	intronic	*LRRC6*	0.609	0.000
	chr8:133833783	rs150145422	C	T	0.010		-0.208	0.035	4.70E-09	intronic	*PHF20L1*	0.598	0.001
	chr8:133848532	rs117764941	G	A	0.018		-0.257	0.027	7.76E-22	intronic	*PHF20L1*	1.000	0.001
	chr8:133869543	rs78404781	A	G	0.018		-0.255	0.027	1.13E-21	intergenic	*PHF20L1;TG*	0.995	0.001
**top hit chr8:133848532 rs117764941**	**chr8:133881996**	**rs116340633**	**G**	**A**	**0.018**	**G67S**	**-0.255**	**0.027**	**1.07E-21**	**nonsynonymous**	***TG***	**0.991**	**0.001**
	chr8:133897237	rs77878855	C	T	0.010		-0.211	0.035	2.14E-09	intronic	*TG*	0.587	0.001
	chr8:133899051	rs116254142	G	A	0.018	V478V	-0.254	0.027	9.73E-22	synonymous	*TG*	0.976	0.001
	chr8:133930184	rs117074997	T	C	0.018		-0.256	0.027	8.88E-22	intronic	*TG*	0.938	0.001
	chr8:133950217	rs146593856	G	T	0.018		-0.251	0.027	5.80E-21	intronic	*TG*	0.909	0.001
	chr8:133959042	rs141409935	G	A	0.010		-0.186	0.036	3.19E-07	intronic	*TG*	0.497	0.000
	chr8:133964191	rs150123915	G	A	0.018		-0.252	0.027	5.03E-21	intronic	*TG*	0.906	0.001
	chr8:133975600	rs118187271	G	A	0.018		-0.246	0.027	6.58E-20	intronic	*TG*	0.892	0.001
	chr8:133529888	rs77148314	G	A	0.037		-0.108	0.014	9.22E-15	intergenic	*KCNQ3;HPYR1*	0.001	0.293
	chr8:133562885	rs192230248	C	T	0.028		-0.144	0.016	1.39E-19	intergenic	*KCNQ3;HPYR1*	0.001	0.512
	chr8:133588839	rs77709138	G	C	0.025		-0.161	0.016	1.25E-22	intronic	*LRRC6*	0.001	0.596
	chr8:133602619	rs116961073	G	A	0.028		-0.148	0.015	3.46E-23	intronic	*LRRC6*	0.001	0.603
**top hit chr8:133771635 rs118039499**	chr8:133771635	rs118039499	A	C	0.023		-0.181	0.017	6.30E-27	intronic	*TMEM71*	0.001	1.000
	chr8:133849655	rs75143612	G	C	0.024		-0.172	0.016	7.58E-27	intronic	*PHF20L1*	0.001	0.916
	chr8:133881471	rs78775620	C	T	0.024		-0.170	0.016	1.63E-26	intronic	*TG*	0.001	0.916
	**chr8:133883671**	**rs114322847**	**C**	**T**	**0.024**	**P118L**	**-0.170**	**0.016**	**1.87E-26**	**nonsynonymous**	***TG***	**0.001**	**0.916**
	chr8:133883905	rs79676842	G	T	0.024		-0.170	0.016	1.97E-26	intronic	*TG*	0.001	0.916
	chr8:133999440	rs74591804	A	C	0.024		-0.095	0.019	8.56E-07	intronic	*TG*	0.000	0.212

*Effect is with regard to Alt allele and the unit is one standard deviation (s.d.) of TSH levels
**Allele frequncy is with regard to Alt allele
***SE: standard error
