# Supplementary material for: GWAS of thyroid stimulating hormone highlights pleiotropic effects and inverse association with thyroid cancer
Source: Nat Commun. 2020 Aug 7;11:3981. doi: 10.1038/s41467-020-17718-z (PMC7414135; doi:10.1038/s41467-020-17718-z)
Supplement: Supplementary file 3 — Descriptions of Additional Supplementary Files [file 41467_2020_17718_MOESM3_ESM.pdf]

Descriptions of Additional Supplementary Files

Supplementary Data 1: Novel TSH loci that have been identified in the meta-analysis of the HUNT study, the MGI biobank and the ThyroidOmics consortium

Supplementary Data 2: Previously reported TSH loci that are significant in the meta-analysis of HUNT, MGI and ThyroidOmics with P-value < $5x10^{-8}$.

Supplementary Data 3: Fine-mapping results reported by SuSiE for 72 TSH loci.

Supplementary Data 4: Tissues in which TSH genes are likely to be highly expressed identified by DEPICT.

Supplementary Data 5: Gene prioritization by DEPICT based on functional similarity to other genes among TSH loci.

Supplementary Data 6: Gene set enrichment test results among TSH loci from DEPICT.

Supplementary Data 7: TSH index variants that are significantly associated (P-value < $5x10^{-8}$) with human diseases (among 1,283 binary phenotypes) in the UK Biobank.

Supplementary Data 8: TSH index variants that are significantly associated (P-value < $5x10^{-8}$) with other quantitative traits in the UK Biobank.

Supplementary Data 9: Association results for thyroid cancer and T4 for all TSH index variants

Supplementary Data 10: The association results with TSH and T4 of 11 previously reported variants for thyroid cancer.

Supplementary Data 11: Phenome-wide association results of the TSH PGS in the UK Biobank.

Supplementary Data 12: The variance of phenotypes (Nagelkerke's r^2) explained by TSH PGS for 1,283 phenotypes based on 280,943 unrelated samples with white British ancestry in the UK Biobank

Supplementary Data 13: Phenome-wide association results of the TSH PGS in FinnGen release 3. Effect is in the unit of one standard deviation (s.d.) of TSH PGS in the FinnGen release 3.

Supplementary Data 14: Two-sample Mendelian Randomization between TSH and thyroid cancer.

Supplementary Data 15: Results of sensitivity check from GWAS of TSH on HUNT participants with TSH levels in normal range and on HUNT participants younger than 50 years old, respectively**.**

Supplementary Data 16: Sensitivity analysis - Two-sample Mendelian Randomization between TSH (GWAS of TSH was conducted using HUNT with participants younger than 50 yrs old) and thyroid cancer

Supplementary Data 17: Two-sample Mendelian Randomization between TSH and goiter
