# Supplementary material for: GWAS of thyroid stimulating hormone highlights pleiotropic effects and inverse association with thyroid cancer
Source: Nat Commun. 2020 Aug 7;11:3981. doi: 10.1038/s41467-020-17718-z (PMC7414135; doi:10.1038/s41467-020-17718-z)
Supplement: Supplementary file 21 — Reporting Summary [file 41467_2020_17718_MOESM21_ESM.pdf]

nature research

Corresponding author(s): Bjørn Olav Åsvold, Wei Zhou

Last updated by author(s): Jun 21, 2020

Reporting Summary

Nature Research wishes to improve the reproducibility of the work that we publish. This form provides structure for consistency and transparency in reporting. For further information on Nature Research policies, see Authors & Referees and the Editorial Policy Checklist .

Statistics

For all statistical analyses, confirm that the following items are present in the figure legend, table legend, main text, or Methods section.

n/a	Confirmed	
☐	☒	The exact sample size (*n*) for each experimental group/condition, given as a discrete number and unit of measurement
☒	☐	A statement on whether measurements were taken from distinct samples or whether the same sample was measured repeatedly
☐	☒	The statistical test(s) used AND whether they are one- or two-sided<br>*Only common tests should be described solely by name; describe more complex techniques in the Methods section.*
☐	☒	A description of all covariates tested
☐	☒	A description of any assumptions or corrections, such as tests of normality and adjustment for multiple comparisons
☐	☒	A full description of the statistical parameters including central tendency (e.g. means) or other basic estimates (e.g. regression coefficient) AND variation (e.g. standard deviation) or associated estimates of uncertainty (e.g. confidence intervals)
☐	☒	For null hypothesis testing, the test statistic (e.g. *F*, *t*, *r*) with confidence intervals, effect sizes, degrees of freedom and *P* value noted<br>*Give P values as exact values whenever suitable.*
☐	☒	For Bayesian analysis, information on the choice of priors and Markov chain Monte Carlo settings
☐	☒	For hierarchical and complex designs, identification of the appropriate level for tests and full reporting of outcomes
☐	☒	Estimates of effect sizes (e.g. Cohen's *d*, Pearson's *r*), indicating how they were calculated

Our web collection on statistics for biologists contains articles on many of the points above.

Software and code

Policy information about availability of computer code

Data collection	We did not use software for data collection
Data analysis	We ran single-variant association tests using SAIGE v0.35.6 and gene-based association tests using SAIGE-GENE v0.35.6. We ran meta-analysis using METAL (version date: 2011-03-25). We fine-mapped TSH loci using susieR_0.8.0. We annotated genetic variants using ANNOVAR (version date: 2017-07-17). We ran DEPICT v1 for enrichment analysis. We ran conditional analysis using GCTA v1.93.0beta and run LD-pruning using PLINK v1.9. We estimated the Nagelkerke's r2 using the R library rcompanion(v2.3.0) .The version of R used for data analysis is 3.6.3.

For manuscripts utilizing custom algorithms or software that are central to the research but not yet described in published literature, software must be made available to editors/reviewers. We strongly encourage code deposition in a community repository (e.g. GitHub). See the Nature Research guidelines for submitting code & software for further information.

Data

Policy information about availability of data

All manuscripts must include a data availability statement. This statement should provide the following information, where applicable:

- Accession codes, unique identifiers, or web links for publicly available datasets
- A list of figures that have associated raw data
- A description of any restrictions on data availability

Data generated or analyzed during this study is available from the corresponding authors upon reasonable request. Data generated or analyzed during this study are available from the corresponding authors upon reasonable request. Meta-analysis TSH summary statistics are available at http://csg.sph.umich.edu/willer/public/TSH2020/.

Field-specific reporting

Please select the one below that is the best fit for your research. If you are not sure, read the appropriate sections before making your selection.

- [x] Life sciences
- [ ] Behavioural & social sciences
- [ ] Ecological, evolutionary & environmental sciences

For a reference copy of the document with all sections, see nature.com/documents/nr-reporting-summary-flat.pdf

Life sciences study design

All studies must disclose on these points even when the disclosure is negative.

Sample size	Sample size was determined as the sum of all contributing cohorts. No statistical method was used to determine sample size.
Data exclusions	Individuals with thyroid disorders based on self-report, blood tests and cancer registry data (7.34%) were excluded from the HUNT data set. In the MGI data set, 5,918 individuals with any thyroid disorders were excluded based on the ICD9 and ICD10 codes mapped to PheCodes52 193 (thyroid cancer), 244 (hypothyroidism), 245 (thyroiditis) and 246 (other disorders of thyroid). TSH summary statistics of meta-analysis reported by the ThyroidOmics consortium were directly used without data exclusion.
Replication	We did not proceed to the replication of our findings as we followed the suggestions of the reviewers' to increase the power of discovery by meta-analyzing HUNT, MGI and ThyroidOmics together.
Randomization	No participants were randomized in this study as we analyze observational data in our study.
Blinding	Blinding was not relevant to our study as there were not experiments done separately on case/control groups.

Reporting for specific materials, systems and methods

We require information from authors about some types of materials, experimental systems and methods used in many studies. Here, indicate whether each material, system or method listed is relevant to your study. If you are not sure if a list item applies to your research, read the appropriate section before selecting a response.

Materials & experimental systems

n/a	Involved in the study	
[ ]	[x]	Antibodies
[ ]	[x]	Eukaryotic cell lines
[x]	[ ]	Palaeontology
[x]	[ ]	Animals and other organisms
[ ]	[x]	Human research participants
[x]	[ ]	Clinical data

Methods

n/a	Involved in the study	
[x]	[ ]	ChIP-seq
[x]	[ ]	Flow cytometry
[x]	[ ]	MRI-based neuroimaging

Antibodies

Antibodies used	For Western blotting of thyroglobulin protein secreted into serum-free media bathing 293T cells, anti-thyroglobulin rabbit polyclonal antibodies were used. Identical results have been obtained using mAb: Thyroglobulin Antibody (D-9): sc-365997 from Santa Cruz Biotechnology (URL: https://www.scbt.com/p/thyroglobulin-antibody-d-9
Validation	Both positive (transfected) and negative (untransfected) controls hav been used: a positive control is secretion of endogenous thyroglobulin from the rat thyroid cell line PCCL3, and a negaive control is absence of secretion of thyroglobulin from untransfected 293T cells.

Eukaryotic cell lines

Policy information about cell lines

Cell line source(s)	293T cell were obtained from the ATCC 293T (ATCC® CRL-3216™).
Authentication	When used by us, the cell line was not authenticated.
Mycoplasma contamination	We confirm that our cells were free of mycoplasma contamination; validated as negative by PCR.
Commonly misidentified lines (See ICLAC register)	No.

Human research participants

Policy information about studies involving human research participants

Population characteristics	HUNT: Participants included both male and female individuals (54.4% are females). The mean age at TSH measurement is 51.79 years. The mean TSH in HUNT is 1.659 mU/L and SD is 1.036 mU/L. MGI: 10,085 samples with TSH measurements included in this study have both male and female individuals (53.4% are females). The mean and SD for TSH in MGI are 1.914 mU/L and 1.175 mU/L, respectively. The mean age at TSH measurement is 55.90 years.
Recruitment	HUNT participants were recruited from a specific region in Norway as part of a population-based health cohort. MGI participants were recruited from pre-surgical encounters at Michigan Medicine (University of Michigan Health System). ThyroidOmics results were taken from previously published study.
Ethics oversight	MGI was approved with the IRB of the University of Michigan. HUNT was approved by the Data Inspectorate and the Regional Ethics Committee for Medical Research in Norway.

Note that full information on the approval of the study protocol must also be provided in the manuscript.
